# Facts for the Curious

**Published:** 1881-09

**Authors:** 


### FACTS FOR THE CURIOUS.

Among the Gauls cutting off the hair was inflicted as a punishment.

Cambric was originally manufactured in Cambrey—hence its name.

Aconite is said to be named from Acone, a place in the Crimea, famous for its poisonous herbs.

A magnificent oak stands on the farm of Major McDowell, near Frankfort, Ky. It is nearly 120 feet in height, and seven men, touching their fingers, tip to tip, could just encircle it at the height of their shoulders.

In a recent sun disturbance a protuberance was thrown up from the surface which was 235,000 miles long, but in a few hours it subsided to only 18,000 miles.

For a healthy adult man the average quantity of food required during twenty-four hours is sixteen ounces of bread, three and a half ounces of butter, and fifty-two fluid ounces of water.

The Romans attached great significance to the color of the hair. Auburn or light hair was considered most desirable, and long before the time of Judas red hair was regarded with great disfavor.

The Glasgow *News* gives an account of a very queer funeral at Sheffield, Eng. The person interred was a deaf mute, and the twelve mourners were also deaf and dumb. The funeral ceremony was conducted entirely by signs.

The largest pasture in the world is on the border between New Mexico and Texas. The Indian Territory bounds it on one side and Colorado on the other. On one side there are forty miles of perpendicular rock fence, and yet it will require 200 miles of fencing to inclose it. The owner, Taylor Mandlin, has sown 1,000 tons of oats. He will feed on it 100,000 head of cattle.

A Washington girl has highly interesting hair. Its color used to be a light blonde. Dr. D. W. Prentiss reports to the Smithsonian Institution that he gave her jaborandi, a Brazilian plant, as a cure for blood poisoning. Her hair soon began to darken, and in four months was almost black.

